# Experimental Study of Hardened Young’s Modulus for 3D Printed Mortar

**DOI:** 10.3390/ma14247643

**Published:** 2021-12-11

**Authors:** Szymon Skibicki, Mateusz Techman, Karol Federowicz, Norbert Olczyk, Marcin Hoffmann

**Affiliations:** 1 Faculty of Civil and Environmental Engineering, West Pomeranian University of Technology in Szczecin, al. Piastów 50a, 70-311 Szczecin, Poland; karol.federowicz@zut.edu.pl (K.F.); norbert.olczyk@zut.edu.pl (N.O.); 2 Faculty of Mechanical Engineering and Mechatronics, West Pomeranian University of Technology in Szczecin, al. Piastów 19, 70-310 Szczecin, Poland; marcin.hoffmann@zut.edu.pl

**Keywords:** 3D mortar, 3D concrete, Young’s modulus, elastic modulus, harden property

## Abstract

Few studies have focused on determining the Young’s modulus of 3D printed structures. This study presents the results of experimental investigations of Young’s modulus of a 3D printed mortar. Specimens were prepared in four different ways to investigate possible application of different methods for 3D printed structures. Study determines the influence of the number of layers on mechanical properties of printed samples. Results have shown a strong statistical correlation between the number of layers and value of Young’s modulus. The compressive strength and Young’s modulus reduction compared to standard cylindrical sample were up to 43.1% and 19.8%, respectively. Results of the study shed light on the differences between the current standard specimen used for determination of Young’s modulus and the specimen prepared by 3D printing. The community should discuss the problem of standardization of test methods in view of visible differences between different types of specimens.

## 1. Introduction

The 3D printing of cementitious materials is one of the fastest growing branches of industry in recent years. A significant number of studies have been conducted on the properties of the fresh mix used in the process of printing. The focus is on being able to print bigger structures in a shorter time [1,2,3,4,5,6,7,8,9]. However, the long-term material properties of hardened cementitious composite should also be considered [10,11,12,13,14,15]. Due to multi-layer characteristic of the printed structure, it is necessary to determine properties such as compressive strength, flexural strength, or modulus of elasticity to fully understand the behaviour of the structure as a whole. Those parameters are essential for proper structural designing with 3D printing.

The majority of studies on hardened properties have focused on compressive strength and flexural strength in regard to the anisotropic behaviour of printed elements. Anisotropy is caused by the layered structure of the elements [16,17,18,19,20,21]. Additionally, a significant number of published research articles have determined the properties on standard samples, proving the negative impact of the printing process on the final values of compressive strength and flexural strength [17,18,22,23,24,25,26]. However, several studies implicate that the printed specimens have higher strength than the standard ones [27,28]. Besides the mechanical strength, one of the key characteristics in structural design is the modulus of elasticity and the Poisson ratio. A handful of studies have determined the stiffness of the fresh mix, which is directly correlated to buildability, a major property in 3D printing [2,29,30,31,32,33,34,35,36,37]. Unfortunately, there are not many studies that undertake the topic of modulus of elasticity and Poisson ratio in 3D printed, multilayer hardened structures.

Based on extensive search on Scopus and Web of Science databases (keywords: 3D concrete, 3D mortar, Young’s modulus, elastic modulus, and hardened property), the most related studies have been selected. A detailed analysis of the results has shown that only several studies have tried to determine the modulus of elasticity of hardened concrete.

Zhang et al. [21] has conducted a study of modulus of elasticity on hardened concrete by cutting out prism specimen (100 mm × 100 mm × 300 mm) from a multilayer printed structure. Due to low height of the prepared print, the load during the test was applied to specimens in the printing direction. The compressive strength of samples tested in the study was 35.12 MPa, the modulus of elasticity E = 36.6 GPa, and the Poisson ratio was 0.28. The mix prepared for the study had ratio of sand (<1 mm) to cement of *S/C* = 1.2 and *W/C* = 0.35. The authors added 2% of nanoclay and 2% of silica fume. Unfortunately, the study does not present the results of standard samples; therefore, it is impossible to determine the impact of 3D printing.

Van der Heever et al. [38] has conducted a study of the modulus of elasticity for samples cut out of a printed structure. Cylindrical samples (d = 28 mm, h = 60 mm) were taken in a perpendicular and longitudinal direction to the layer orientation. The height of the layer in the study was assumed to be 10 mm. The mix had the *w/c* = 0.46 (*w/b* = 0.32). Mix was made on CEM II 52.5N cement with the maximum size of the aggregate being 4.75 mm, and additional polypropylene fibers (l = 6 mm). Obtained results of the Young’s modulus were similar regardless of the specimen orientation (perpendicular E_mod_ = 21.6, CoV = 6.2%, longitudinal E_mod_ = 21.9 GPa, and CoV = 4.8%). The article does not present the comparison to standard samples; the authors only refer to the theoretical values of Young’s modulus based on the compressive strength of printed specimen. The difference according to authors reached 8 GPa and was seen as a result of differences in porosity [39,40].

Wu et al. [41] has used a nanoindentation at micro-scale tests and the representative volume element methods (Monte Carlo), as well as results found in the literature, to obtain the results of Young’s modulus for a 3D printed structure. The authors have obtained in their theoretical calculations a Young’s modulus of 29.17 GPa (Poisson’s ration of 0.2), while the initial results taken from literature have shown a E_mod_ = 30 GPa with Poisson’s ration of 0.22 [42]. It needs to be said that the results were only theoretical and not confirmed experimentally. Individual results of linear elastic constitutive matrix have huge discrepancies, particularly the mean values of the components of effective elasticity matrix. Additionally, the simulation omitted the interlayer transition zone, which can play a major role in the change of mechanical characteristics of 3D printed concrete [43,44,45,46].

Zahabizadeh et al. [16] has designed two mixes on a CEM I 42.5 (w/b = 0.31), with an aggregate of up to 1 mm and the addition of fly ash (FA). The mixes had different ratios of cement and FA. Nominal compressive strengths of tested mixes were 58.0 MPa and 75.6 MPa. The authors have determined the modulus of elasticity on molded prism samples (50 mm × 50 mm × 100 mm) and samples cut out from printed structure. Both specimen types were cut to the size of 40 mm × 40 mm × 80 mm. The Young’s modulus was tested in two directions: perpendicular and longitudinal, to the layer orientation. Obtained results for studied mixes ranged from 27 GPa to 36 GPa. The biggest difference in Young’s modulus between molded and printed samples was 8%, while for compressive strength the discrepancy increased to 18%. The values of Young’s modulus and compressive strength were 8% and 18% higher in the longitudinal direction, respectively.

Feng et al. [47] has analyzed the Young’s modulus for powder bed fusion prints. In this method, the printed structure has a support that allows for a better compaction between the layers. The determination was performed for a cubic specimen with sides of 70.7 mm and 50 mm. The height of a single layer was 0.0875 mm. The Young’s modulus determined on cubic 70.7 mm specimen was tested in longitudinal and lateral directions. The results of the determination were 3.6 GPa and 1.9 GPa, respectively.

To summarize, only several articles take on the topic of determination of Young’s modulus in 3D printed structures. Moreover, none of above-mentioned studies directly refer to the influence of layer number on the results of Young’s modulus. Only Van der Heever et al. [38] has performed the tests on the cylindrical samples, where the stress distribution is easy to determine and can be compared to samples made in accordance with European standards [48]. This approach allows one to obtain values that can be used in real-life structural design. The authors of that study did not compare the results to standard, molded samples. Other researchers chose prism specimens [16,21,47], which cannot be directly correlated to samples determined by the standards. In some cases, the specimens were not even compared to molded samples [21,47].

The aim of this study is to determine the influence of number of layers and specimen size on the values of Young’s modulus in printed structures. The research determines the relation between the specimen size and method of preparation on the values of Youngs modulus. The values are compared to standards samples, which will allow one to implement them for the purpose of structural design.

## 2. Materials and Methods

### 2.1. Materials

The mix used in the study was previously presented in [3,32,49]. The water/binder ratio was 0.3. Total binder amount is 829 kg/m^3^. The binder in mix consists of 70% of cement (CEM I 52.5R), 20% of fly ash and 10% of silica fume. The fly ash used in the study was obtained from a local coal power plant. The aggregate was a fine natural sand of 0–2 mm. A polycarboxylate powder water-reducing admixture was used to adjust the rheological properties of the mix.

**Figure 1:**
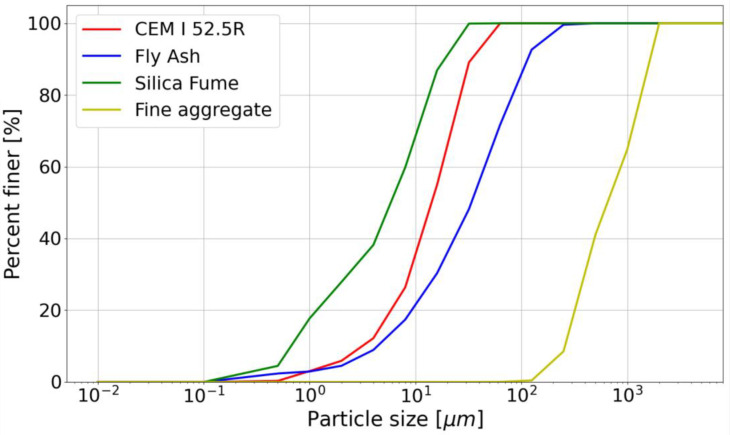
Particle size distribution curve.

The chemical compositions of the materials used are shown in Table 1. Particle size distribution for used materials is presented in Figure 1. The curves for cement, silica fume and fly ash were obtained by laser diffraction method and for fine aggregate by sieve method. Mix composition is given in Table 2

### 2.2. Experimental Procedure

The experimental procedure was designed to compare the results of Young’s modulus of samples prepared in different way. The study also tries to determine if the size of printed specimen, which results in different number of layers and layer locations, influences this material property. The study determines the correlation of layer number on the mechanical performance of 3D printed elements.

#### 2.2.1. Mixing Procedure

A standard 110 L planetary mixer (Controls, Milan, Italy) was used in this study. All dry materials, including cement, mineral additives, sand and powder water-reducing admixture (PCA) were initially mixed for 5 min. Then, three quarters of the water was added to the mixer. The mix was mixed for 5 min, then the homogeneity of the mix was evaluated. The remaining water was added to improve the workability of the mix.

Mix preparation and printing were made in a laboratory at a temperature of 20 °C (±2 °C) and relative humidity of RH = 55% (±5%).

#### 2.2.2. Fresh Properties

For the purpose of this study, a constant slump flow of 160 mm ± 10 mm was assumed. The slump was determined 15 min after adding the water in accordance with the standard [50]. Similar assumptions for suitability of mixes for 3D printing were proposed in other studies [3,31,51,52,53,54]. The mix was then pumped to obtain mix for the determination of the buildability.

The buildability was determined in an unconfined uniaxial compression test. A similar test can be found in [17,30,55,56]. The test uses cylindrical Φ60 mm × 35 mm samples. The test allows one to obtain the stress–strain relationship for the examined mix. The test results determine the green strength at failure and Young’s Modulus of the mix. This approach allows one to find mix load-bearing and deformation behavior after deposition. Test was performed at a constant displacement rate of 30 mm/min. The test was performed between 15 and 30 min after adding water to the dry ingredients. The specimens were formed immediately before testing and compacted manually. The test was performed three times.

**Figure 2:**
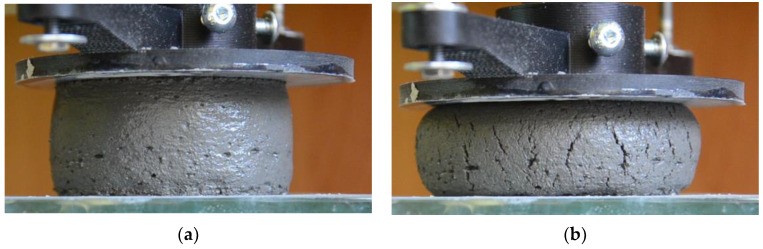
Specimen during unconfined uniaxial compression test: (**a**) specimen at the beginning of the test; (**b**) specimen after the test.

The deformation of the specimen during the test was recorded by LVDT (Linear Variable Differential Transformer) displacement transducers (0.01 mm accuracy) (HBM, Darmstadt, Germany) connected to the HBM QuantumX strain gauge bridge (MX840A, HBM, Darmstadt, Germany). The detailed description of the testing bench can be found in [23]. The specimen during the test is presented in Figure 2.

The mix was then initially printed to visually evaluate the path quality. The quality of path was evaluated by following points:

- The sizes of path should be constant;
- The global deformations of path are unacceptable;
- The printed layer must be free of surface defects and cracks (only small minor cracks and defect can be acceptable).

By meeting the mentioned criteria, the mix was accepted for preparation of specimen.

#### 2.2.3. Specimen Preparation

Four different methods of preparing samples were considered in this study. Typical cylindrical specimen prepared for ordinary concrete do not reflect the way how the printed concrete works. Therefore, the chosen methods not only address the material properties but also various ways of preparing the samples, which could have a potential application in in-situ testing of printed structure.

The first type was the reference, standard Φ15 cm × 30 cm cylindrical specimen that was mold-casted. The samples were prepared in a conventional way in accordance with EN 12390-3 [57] and EN 12390-13 [48]. The specimens were left for 24 h in laboratory before demolding and further curing.

**Figure 3:**
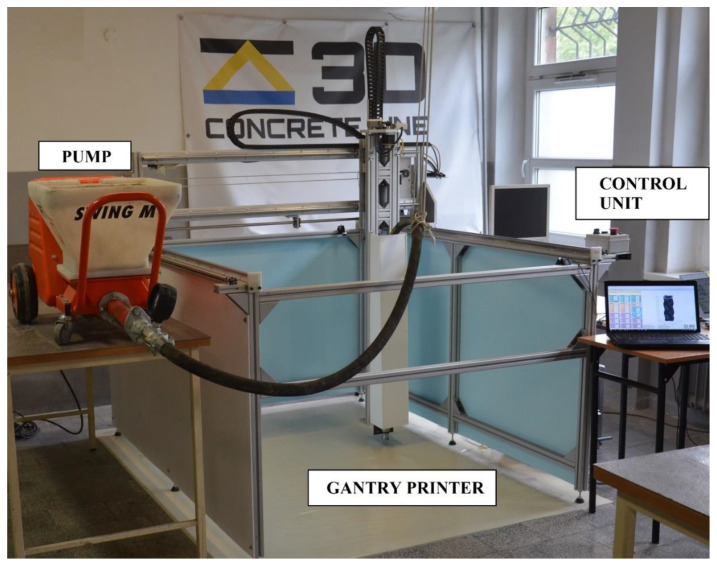
Printing setup used in the study.

The 3D printed specimens were prepared using the additive manufacturing extrusion method [6,58]. For this purpose, a gantry printer (3DoF) with a concrete rotor-stator pump was used. The system is controlled by a G-Code. For all printed specimen, the constant deposition rate of 0.75 L/min was assumed. Depending on the type of specimen described below, the printing speed and pump output was adjusted. The printing setup is presented in Figure 3.

**Figure 4:**
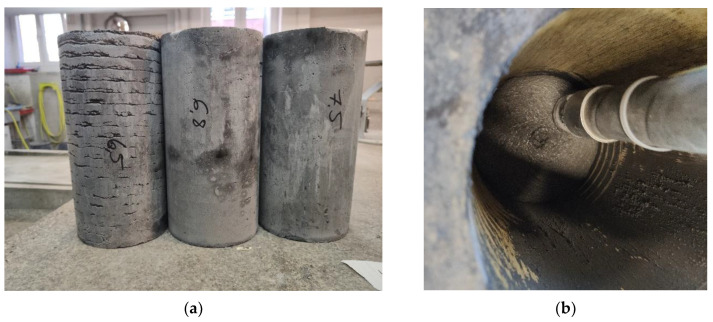
Samples printed in the mold: (**a**) evaluation of pump output for samples printed into the mold; (**b**) view of the printing process.

Second type of specimens was prepared by printing within the typical Φ15 cm × 30 cm mold. The printing path was generated based on a spiral to ensure proper infill of the mold. The pump parameters were set to obtain full cross-section of the specimen. Evaluation of proper pump output and the sample during printing within the mold is presented in Figure 4. The samples were printed directly from a Φ25 mm hose, rigidly fixed to the printer. Similar to the standard samples, the specimens were left for 24 h in laboratory before demolding and further curing.

**Figure 5:**
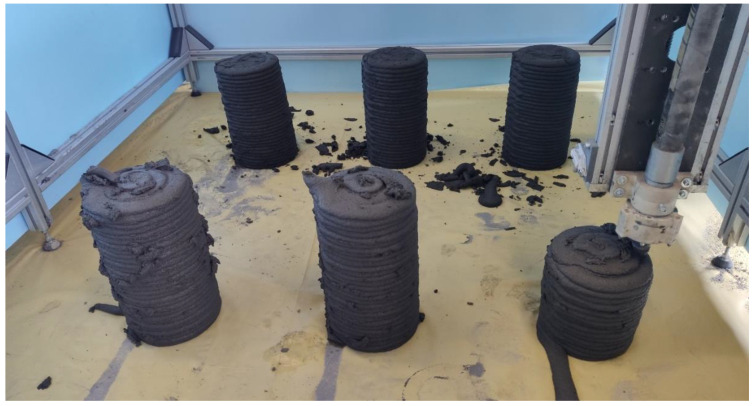
Printing of concrete columns.

Third type of specimens was created by printing columns (as presented in Figure 5). The printing was performed using a Φ25 mm nozzle with a flat end, to provide as even a surface of the layer as possible. The columns had an outer diameter of 150 ± 5 mm. The outer sides of the specimen were not trowelled. The loose, excessive chunks of the fresh mix were gently removed from the specimen immediately after printing. The samples were then sprayed with a water mist and covered with a PE film for 24 h before being cured in water for the remaining period.

**Figure 6:**
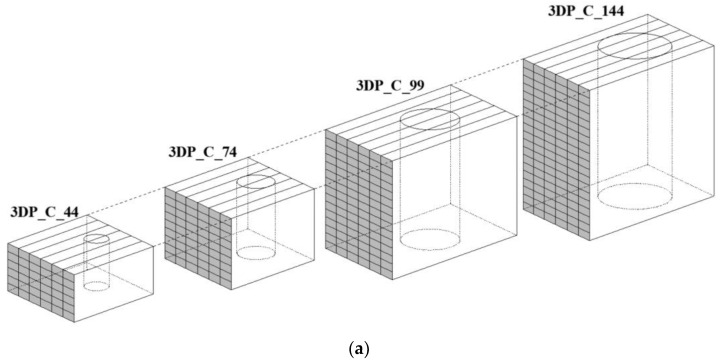

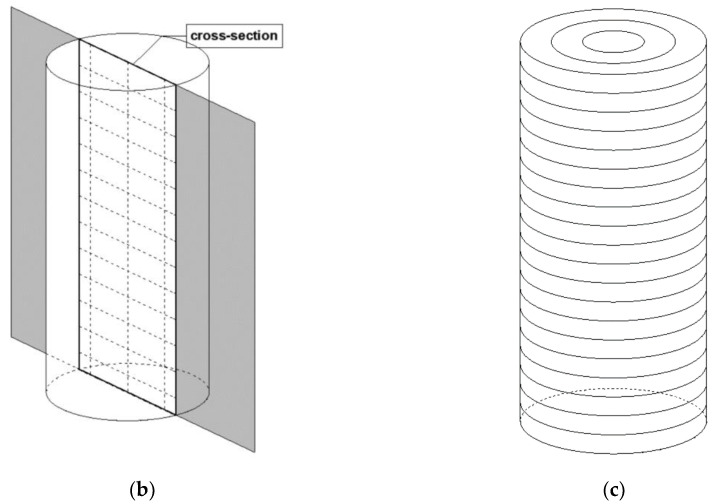
Schematics of samples: (**a**) core drilled from printed structure-plan with layers orientation; (**b**) core drilled from printed structure-example of cross-section; and (**c**) freely 3d printed columns and specimens printed into mold.

The fourth type of specimens was prepared by cutting them from a bigger 3D printed multi-layer block. The initial block was printed using a Φ25 mm nozzle with parallel layers. The printing speed was adjusted to obtain good visual vertical adhesion of the layers. The specimen were cut in four different sizes to determine the influence of the number of layers on the mechanical characteristics. The diameter of cut out samples chosen for the study was 44 mm, 74 mm, 99 mm and 144 mm. The cutting was made using typical diamond saw for concrete and stone. The sizes were chosen based on available core drill sizes. The cut-out samples were then cut to reach the ratio of length to diameter of l/d = 2 ± 0.1. The schematics of drilled samples are presented in Figure 6.

The notation of all samples prepared in the study is as follows:

- STDR—standard mold-casted specimens Φ15 cm × 30 cm;
- 3DP_M—specimens 3D printed into a Φ15 cm × 30 cm mold;
- 3DP_F—freely 3D printed columns approx. Φ15 cm × 30 cm;
- 3DP_C_X—3D printed specimens cut from a block. The X stands for a diameter of the sample in mm.

All specimens after initial 24 h curing time were cured in water at 20 ± 2 °C for additional 26 days before the tests. The samples were then taken out and surface-dried to prepare them for attaching the strain gauges (Techno-Mechanik, Gdańsk, Poland). The specimen was stored for the last 24 h in laboratory conditions. The total curing took 28 days. Notations and sample characteristics were shown in Table 3.

#### 2.2.4. Young’s Modulus and Compressive Strength

For the purpose of compressive strength and Young’s modulus determination, six samples were prepared for each specimen type. The samples were prepared and tested in accordance with [48]. The upper and bottom parts of the specimen were either cut off to obtain even and parallel surfaces or if possible, capped with high-strength fast setting mix.

**Figure 7:**
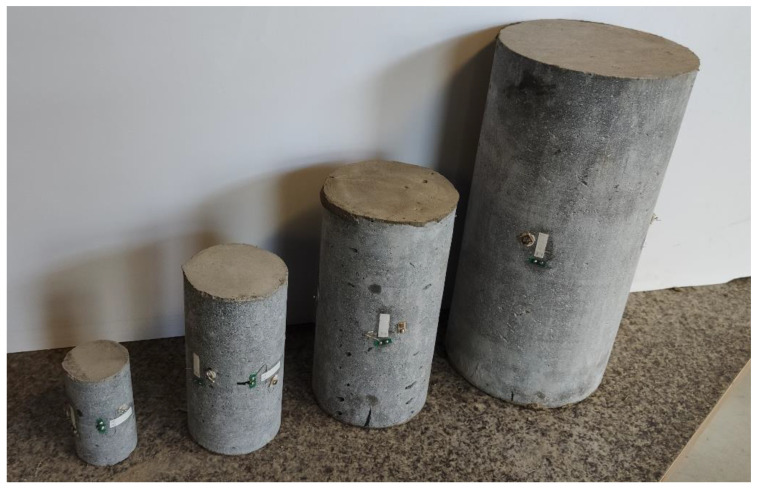
Core drilled specimens with strain gauges installed on them.

The Young’s modulus test was performed in accordance with [48]. Each specimen Φ15 cm × 30 cm was prepared by symmetrically attaching three vertical and two horizontal strain gauges with a base of 75 mm and k-Gauge factor of 2.15. For core drilled specimen smaller strain gauges, with bases of 20 mm, were installed. Examples of samples with strain gauges installed are presented in Figure 7. The strain gauges were connected in a half-bridge. The measurements were recorded by the HBM UPM 60 device (HBM, Darmstadt, Germany).

## 3. Results

### 3.1. Unconfined Uniaxial Compression Test

**Figure 8:**
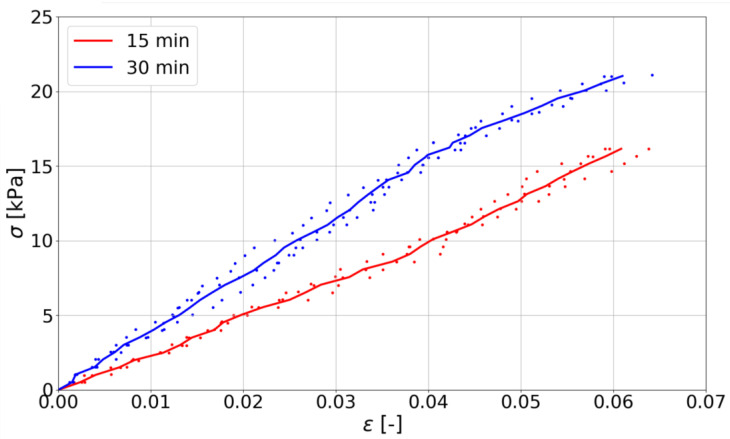
The stress–strain curve for mixes.

The unconfined uniaxial compression test was used to determine the buildability of the mix. The mixes were tested between 15 and 30 min after adding water to the mix, which corresponds to time of printing. Figure 8 presents the stress–strain relation σ(ϵ). The dots represent each individual measurement, while line corresponds to the mean values between two adjacent results.

Table 4 presents the mean values obtained in the study. The green strength and Young’s modulus value, calculated as a slope of σ(ϵ) (see [59]), has been presented.

The mixture during printing can transfer loads between 16.15 kPa and 21.03 kPa. In addition, its stiffness ranges between 263 kPa and 359.32 kPa. Similar results were obtained by Esposito et al. [52], where the compressive strength (green strength) at 15 to 30 min was in the range of 11.64 kPa to 26.04 kPa depending on the type of mixture and test method. The Young’s modulus in their study was between 252 kPa and 488 kPa. Wolfs et al. [60] have obtained strength in the range of 6.99 kPa to 10.87 kPa and Young’s modulus in the range of 54.42 kPa to 98.52 kPa. Ding et al. [34] obtained compressive strength in the range of 9.51 to 45 kPa and Young’s modulus between 29 kPa and 280 kPa. In summary, the values obtained in this study can be considered as correct and meeting the requirements for 3D printed mixes. Results are corroborated by other studies [3,23,29,34,52,59,60].

### 3.2. Young’s Modulus and Compressive Strength

Determination of the mechanical parameters of studied samples is presented in Table 5. Mean values of compressive strength $f_{cm}$, Young’s modulus $E_{cm}$ and Poisson’s ratio $\vartheta_{cm}$ with coefficient of variations CoV are given. Additionally, a percentage strength relation to standard specimens STDR is given calculated based on (1):

(1) $$X_{change}=\frac{X_{cm}-X_{st}}{X_{st}}\cdot 100\%$$

where:

- $X_{change}$—percentage change ($E_{change}$–for Young’s modulus, $f_{change}$–for compressive strength, $\vartheta_{change}$–for Poisson’s ratio);
- $X_{st}$—mean value obtained for standard specimens;
- $X_{cm}$—mean value obtained on a specific specimens type.

**Figure 9:**
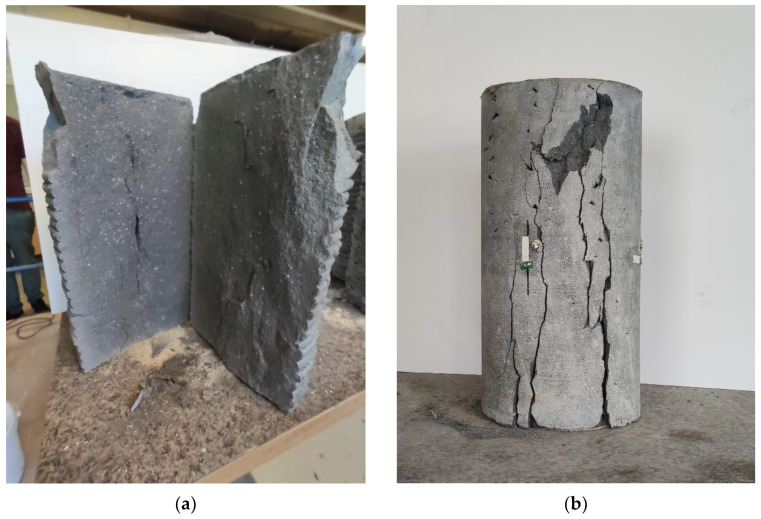
Failure mechanism of specimen: (**a**) freely printed column 3DP_F; (**b**) specimen cut out 3DP_C_144.

**Figure 10:**
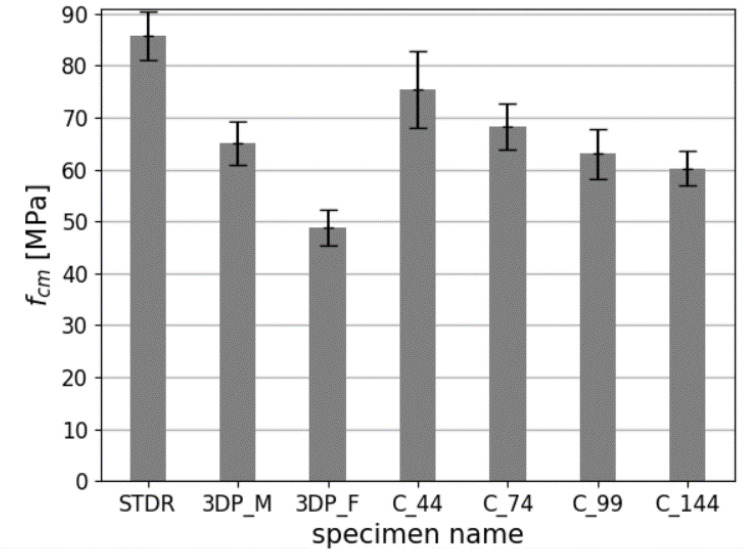
Comparison of the compressive strengths of studied specimens.

**Figure 11:**
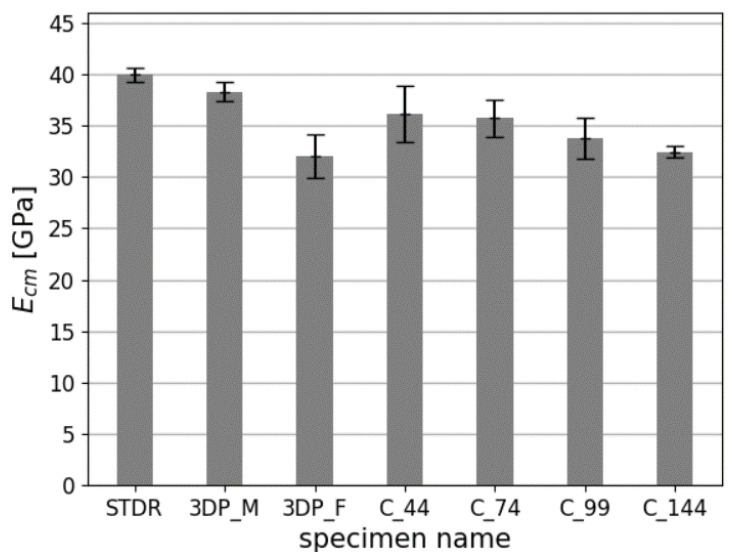
Comparison of the Young’s modulus of studied specimens.

**Figure 12:**
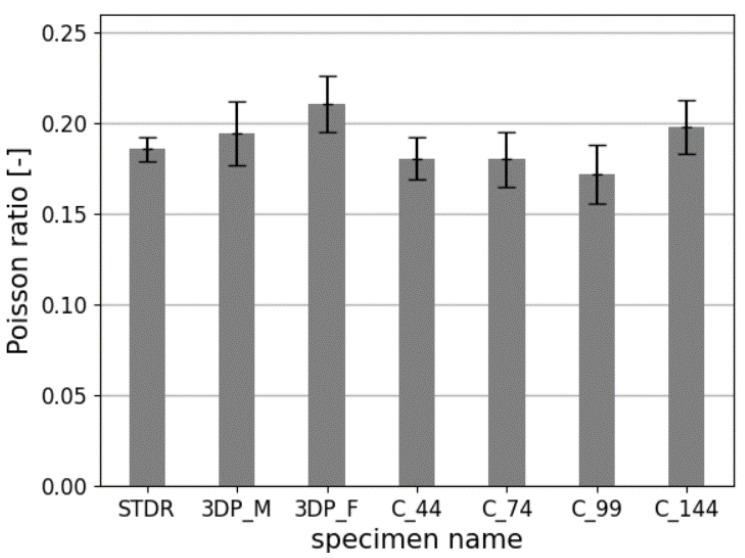
Comparison of the Poisson’s ratios of studied specimens.

The failure mechanism of studied samples is presented in Figure 9. Comparison of results is presented in Figure 10, Figure 11 and Figure 12.

In case of the compressive strength, the highest was achieved by the standard samples (STDR), which seems to be a proper result considering other publications on 3D printing where standard and printed samples where compared [18,22,24,25,61]. The reduction in strength for printed samples ranged from 12% to 43.1%. The lowest compressive strength value was achieved by the 3DP_F specimen (printed cylindrical specimen without any lateral support). For this specimen, the reduction in strength relative to the standard specimen was the greatest, as much as 43.1%. This can also be considered correct because this sample was printed without any side support, resulting in worse compaction at the interlayer zone [19,39,40]. Other samples had either lateral support in the form of a mold (3DP_M samples) or in the form of surrounding layers of printed material-lateral elastic support (samples 3DP_C_44 to C_144). The strength reduction for samples other than the 3DP_F is between 12% and 29.8%.

For Young’s modulus, the difference between the results is less visible. The difference between freely printed samples (3DP_F) and STDR samples reached 19.8%. The highest results of the Young’s modulus were observed for the STDR samples (E = 39, 93 GPa).

No clear correlation between the method of preparing the samples and Poisson’s ratio was observed. The values for all samples were between 0.17 and 0.21, corresponding to a −13.3% to +7.5% change compared to STDR samples. As the CoV for all specimens is rather low, the values obtained in this study concur with the EN 1992-1-1 [62] standard, which assumes the Poisson’s ratio of $\vartheta_{cm}=$ 0.2.

**Figure 13:**
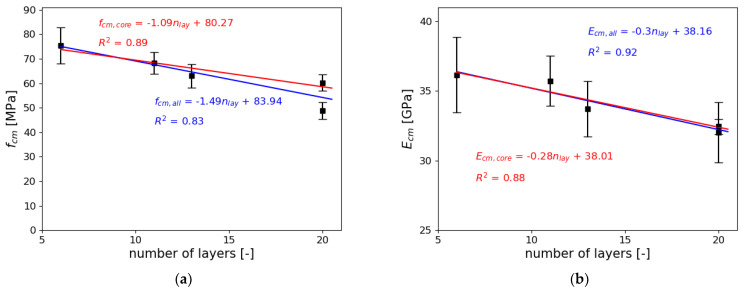
The correlation between the number of layers and (**a**) compressive strength, (**b**) Young’s modulus.

Figure 13 presents the statistical correlation between the number of layers, and the compressive strength and Young’s modulus of specimen. The analysis was performed for the samples cut from a bigger block (3DP_C_X) and a freely printed column (3DP_F), to exclude the possible compaction of layers that could occur in samples printed in the mold.

A linear regression was applied to analyze the results of this study. Analysis was performed for the compressive strength and Young’s modulus. The linear regression was calculated for two groups of samples:

- $f_{cm,all}$ and $E_{cm,all}$—all cut out samples 3DP_C_X and 3DP_F
- $f_{cm,core}$ and $E_{cm,core}$—only cut out samples, excluding 3DP_F

Those two groups were chosen to determine if the sample preparation method (freely printed column or core drilled samples) for 3D printed concrete influences the mechanical properties. Figure 13 presents also the value of the R^2^ (coefficient of determination) as well as standard deviation of the results.

Figure 13a presents the analysis of the results for the compressive strength obtained in the study. The compressive strength of samples decreases with the increase of the number of layers. As seen in the linear regression for all samples ($f_{cm,all}$), the coefficient of determination R^2^ = 0.83. This means that the correlation of the results is not satisfactory. The main reason behind it is the difference between the results of the biggest core drilled samples (3DP_C_144) and the freely printed samples (3DP_F). The latter has a significantly lower mean compressive strength, which results in the decrement of the R^2^ value. Freely printed samples do not have any lateral support, whereas the core drilled samples were initially restricted by surrounding layers. In case of the second group of samples, where the 3DP_F was excluded, the value of R^2^ was 0.89, which is much closer to the value considered as strong correlation of the results.

Figure 13b presents the results of the analysis of Young’s modulus. As in the analysis of the compressive strength, the Young’s modulus decreases with the increase of the number of layers. This confirms the assumption that mechanical material properties will similarly change with the change of layer number. Looking at the results for the biggest printed samples (3DP_F and 3DP_C_144), the differences in the values of Young’s modulus were insignificant and are within the values of CoV. The value of the coefficient of determination for all printed samples ($E_{cm,all}$) was R^2^ = 0.92, while only for core printed samples ($E_{cm,core}$) was R^2^ = 0.88. Both values prove the good correlation of the results. It is worth noticing that the percentage differences in the Young’s modulus is close to the values of CoV, which proves that the differences are insignificant.

## 4. Conclusions

The paper presents the influence of number of layers and preparation method on the values of Young’s modulus of 3D printed concrete. Obtained results for 3D printed samples were compared to standard cylindrical samples. The study extends the knowledge on the determination of Young’s modulus for 3D printed structures. Following conclusions have been drawn:

- The bigger the specimen, the lower the mechanical performance of cut-out samples.
- The higher the number of layers, the lower the value of compressive strength of printed samples. The strength reduction compared to standard cylindrical sample was the highest for freely printed columns (approximately 43%). The strength reduction was lower for samples printed into a mold or cut out from a bigger printed block.
- The higher the number of layers, the lower the value of Young’s modulus of printed samples. The difference between the biggest printed sample and the standard sample reached 20%.
- Samples printed into a mold or cut out from a bigger printed block had better mechanical performance than freely printed columns. This is caused by lateral restriction of concrete due to either mold itself or surrounding layers.
- The value of Poisson’s ratio for printed samples in this study differed by ±13% from the standard samples.

The article presents different ways of preparing the specimen and compares them. None of the printed specimen came close to the values obtained for a standard specimen. This means that the approach to preparing samples for evaluation of 3D printed elements should be reconsidered. The community needs to determine a single, standards methods for determining material parameters of 3D printed concrete for real-life structural applications. Each of the studied methods of preparing the samples has its disadvantages. Samples printed within a formwork require hose extension and can be bothersome. The samples freely printed as columns have variations of dimensions and do not exactly reflect the deformation that would occur when printing higher structures. The samples cut from a bigger printed block can have changed properties due to the cutting itself.

The results of this study has shown a significant reduction in compressive strength and Young’s modulus of 3D printed structures in regard to standard samples. This shows the importance of including the reduction factors in designing protocols of 3D printed structural elements. It is necessary not only to include the reduction in compressive or flexural strengths that can be found in other studies [18,22,61] but also the reduced values of the Young’s modulus.

## Figures and Tables

**Table 1:** Chemical composition of Portland cement, fly ash and silica fume.

Chemical Composition	CEM I 52.5 R [%]	Fly Ash [%]	Silica Fume [%]
SiO_2_	19.70	54.00	94.00
Al_2_O_3_	4.93	28.40	–
Fe_2_O_3_	2.54	7.30	–
CaO	64.23	3.10	0.30
CaCo_3_	–	–	–
MgO	1.32	2.40	–
SO_3_	2.91	0.40	1.90
Na_2_O	0.12	1.10	–
K_2_O	0.76	2.90	–
Cl^–^	0.07	0.01	0.10
H_2_O	–	–	0.70
Na_2_0eq	0.63	–	–
LOI	–	–	3.00

**Table 2:** Mix composition.

Material	Amount [kg/m^3^]
Cement CEM I 52.5R	580
Fly Ash	166
Silica Fume	83
Aggregate 0–2 mm	1335
Water	200
Water-reducing admixture	1.9

**Table 3:** Notations and sample characteristics.

Group	Type	Diameter [mm]	Height [mm]	Number of Layers
I	STDR	150	300	-
	3DP_M	150	300 ± 10	20 ± 1
	3DP_F	150 ± 10	300 ± 10	20 ± 1
II	3DP_C_44	44	90 ± 10	6 ± 1
	3DP_C_74	74	160 ± 10	11 ± 1
	3DP_C_99	99	200 ± 10	13 ± 1
	3DP_C_144	144	300 ± 10	20 ± 1

**Table 4:** Mechanical properties of 3D printed mix.

Time	Green Strength [kPa]		Young’s Modulus [kPa]	
	Mean [kPa]	CoV [%]	Mean [kPa]	CoV [%]
15 min	16.15	4.30	263.00	3.88
30 min	21.03	3.20	359.32	3.52

**Table 5:** Results of compressive strength, Young’s modulus and Poisson’s ratio determination.

Specimen	*f_cm_* [MPa]	CoV [%]	$f_{change}$ (1)	*E_cm_* [GPa]	CoV [%]	$E_{change}$ (1)	$\vartheta_{cm}$ [–]	CoV [%]	$\vartheta_{change}$ (1)
STDR	85.72	5.5%	-	39.93	1.7%	-	0.19	3.5%	-
3DP_M	65.03	6.5%	−24.1%	38.25	2.4%	−4.2%	0.19	9.2%	4.7%
3DP_F	48.77	7.2%	−43.1%	32.01	6.8%	−19.8%	0.21	7.4%	13.3%
3DP_C_44 (C_44)	75.42	9.8%	−12.0%	36.15	7.5%	−9.5%	0.18	6.5%	−2.8%
3DP_C_74 (C_74)	68.17	6.6%	−20.5%	35.71	5.0%	−10.6%	0.18	8.5%	−3.0%
3DP_C_99 (C_99)	63.01	7.5%	−26.5%	33.70	5.9%	−15.6%	0.17	9.5%	−7.5%
3DP_C_144 (C_144)	60.18	5.5%	−29.8%	32.44	1.7%	−18.8%	0.20	7.5%	6.7%

## Data Availability

The data presented in this study are available upon reasonable request from the corresponding author.
